# Outcomes and safety of intravenous thrombolysis in minor ischaemic stroke: a real-world retrospective cohort study

**DOI:** 10.1186/s42466-026-00552-x

**Published:** 2026-11-10

**Authors:** Nedim Leto, S. C. Ajmi, C. R. Munk, E. Farbu, T. W. Lindner, M. W. Kurz

**Affiliations:** 1 Department of Clinical Medicine, Faculty of Medicine, University of Bergen, Bergen, Norway; 2 The Regional Centre for Emergency Medical Research and Development in Western Norway (RAKOS), Stavanger, Norway; 3 Department of Neurology, Neuroscience Research Group, Stavanger University Hospital, Stavanger, Norway

**Keywords:** Ischaemic stroke, Intravenous thrombolysis, Minor stroke, NIHSS, Functional outcome, Stroke mimic, Symptomatic intracranial haemorrhage

## Abstract

**Background:**

The benefit-risk profile of intravenous thrombolysis (IVT) in minor ischaemic stroke remains controversial. We evaluated real-world outcomes and safety of IVT in a centre with a liberal thrombolysis policy.

**Methods:**

We performed a retrospective cohort study of consecutive adult patients treated with IVT for suspected acute ischaemic stroke with admission NIHSS 0–5 at Stavanger University Hospital between 2008 and 2023. A liberal thrombolysis policy was defined as a treatment threshold that does not exclude patients on the basis of a low NIHSS score or apparently non-disabling symptoms alone. Patients were stratified into NIHSS 0–2 and NIHSS 3–5 subgroups. Outcomes included favourable early neurological status, defined as NIHSS 0–1 within 24 h, functional independence at 90 days (mRS 0–2), poor outcome (mRS 3–6), stroke mimic diagnosis, and symptomatic intracranial haemorrhage (sICH). Multivariable logistic regression analyses were performed in confirmed stroke cases, excluding stroke mimics.

**Results:**

Among 2,008 IVT-treated patients, 1270 (63%) had admission NIHSS 0–5: 744 (59%) had NIHSS 0–2 and 526 (41%) had NIHSS 3–5. Stroke mimics accounted for 335 cases (26%) and were more frequent in the NIHSS 0–2 group than in the NIHSS 3–5 group (29% versus 23%; *p* = 0.041). Among patients with available 90-day mRS, 602/697 (86%) achieved functional independence, with higher rates in the NIHSS 0–2 group than in the NIHSS 3–5 group (93% vs. 76%; *p* < 0.001). sICH occurred in four patients (< 1%), with no events among stroke mimics. In adjusted analyses restricted to confirmed stroke cases, NIHSS 0–2 was associated with higher odds of favourable early neurological status and functional independence compared with NIHSS 3–5.

**Conclusions:**

In this real-world cohort from a centre with a liberal thrombolysis policy, IVT-treated patients with minor stroke symptoms had favourable functional outcomes and very low complication rates of sICH. The contribution of this cohort lies in describing what a liberal thrombolysis policy produces in unselected routine practice, as opposed to the narrowly defined populations enrolled in randomised trials. These findings provide clinically relevant safety data, but they cannot demonstrate efficacy and should be interpreted descriptively in the absence of an untreated comparator group.

**Supplementary Information:**

The online version contains supplementary material available at https://doi.org/10.1186/s42466-026-00552-x.

## Background

Stroke remains a leading cause of death and long-term disability worldwide and imposes a substantial burden on healthcare systems, including in Norway [1, 2]. Among these, minor ischaemic stroke, commonly defined as patients presenting with a National Institutes of Health Stroke Scale (NIHSS) score of ≤ 5, accounts for up to half of all stroke presentations [3, 4]. Although neurological deficits are often mild at initial presentation, a considerable proportion of patients experience persistent disability, including cognitive impairment, language dysfunction, reduced participation in society, and impaired quality of life despite apparently favourable functional recovery [5]. The modified Rankin Scale (mRS) was developed to capture major disability and is relatively insensitive to such residual deficits, which has direct consequences for the assessment of outcome in this population [6]. The optimal management of minor ischaemic stroke remains controversial. Current international guidelines recommend intravenous thrombolysis (IVT) for patients with disabling neurological deficits, whereas IVT is generally not recommended for patients with non-disabling symptoms [7, 8]. However, the distinction between disabling and non-disabling stroke remains clinically challenging and is based on the expected functional consequences of the neurological deficit rather than the NIHSS severity alone [9].

Randomised trials including PRISMS, ARAMIS and TEMPO-2 have suggested that dual antiplatelet therapy (DAPT) may be non-inferior to IVT in selected patients with minor non-disabling stroke, while IVT is associated with an increased risk of symptomatic intracranial haemorrhage [7, 10, 11]. These trials are not directly comparable with one another, or with unselected clinical cohorts, since they differ substantially in comparator treatment, time window and eligibility criteria. However, these studies have been criticised for heterogeneous definitions of non-disabling stroke and limited generalisability to routine clinical practice [9, 12]. In contrast, a recent systematic review and meta-analysis including more than 90,000 patients suggested that IVT may be associated with favourable functional outcomes in patients with minor stroke, highlighting the persistent uncertainty regarding optimal treatment strategies [13].

Given these uncertainties, there is a need for large real-world observational studies to better characterise the outcomes and safety of IVT in an unselected minor stroke patient population. At our comprehensive stroke centre, IVT has historically been administered liberally to patients with mild neurological deficits. This provides a unique opportunity to evaluate the safety and functional outcomes of IVT-treated patients in routine clinical practice.

The aim of this study was to describe the functional outcomes and safety of IVT in patients presenting with minor ischaemic stroke (NIHSS 0–5). Prespecified subgroup analyses compared stroke presentation between patients with very mild (NIHSS 0–2) and mild (NIHSS 3–5) deficits. Outcomes included early neurological improvement, functional independence at 90 days, and rates of symptomatic intracranial haemorrhage (sICH).

## Methods

### Study design and population

We conducted a retrospective cohort study of all consecutive adult patients treated with IVT for suspected acute ischaemic stroke at Stavanger University Hospital (SUH) between January 2008 and December 2023. Patients presenting with minor neurological deficits, defined as an admission NIHSS score of 0–5, were included. Throughout the study period the centre applied what we refer to as a liberal thrombolysis policy: IVT was offered to all patients fulfilling standard eligibility criteria, without an additional severity threshold, so that a low NIHSS score or apparently non-disabling symptoms did not in themselves preclude treatment. The practical consequence of this policy is quantified in national audit data: according to the Norwegian Stroke Register, approximately 60% of patients admitted to our centre with an NIHSS of 0–2 received IVT, compared with 6% at the lowest-treating Norwegian centre [14].

For subgroup analyses, patients were stratified according to baseline NIHSS into very mild deficits (NIHSS 0–2) and mild deficits (NIHSS 3–5). Inclusion was independent of final discharge diagnosis, allowing identification and analysis of stroke mimics. The study was conducted and reported in accordance with the Strengthening the Reporting of Observational Studies in Epidemiology (STROBE) guidelines.

### Setting

SUH is a regional stroke centre serving approximately 392,000 inhabitants across an area of 7,000 km². The hospital works in close collaboration with the regional Emergency Medical Services (EMS). In Norway, the EMS system comprises Emergency Medical Dispatch Centres (EMDCs), which assign diagnostic suspicion and urgency based on emergency calls, and ambulance units that perform on-scene assessment and initiate prehospital stroke management (vital signs, ECG, IV access, and hospital prenotification), ensuring rapid evaluation and transport to hospital.

The centre provides 24/7 acute stroke care, including neurological assessment, neuroimaging, IVT and endovascular treatment (EVT) for patients with large vessel occlusion. The hospital also provides 24/7 neurosurgical services and has a dedicated stroke unit with neurocritical care facilities.

All patients underwent non-contrast computed tomography (CT) to exclude intracerebral haemorrhage and other contraindications before IVT. Additional vascular and perfusion imaging, including CT angiography, CT perfusion, and magnetic resonance imaging (MRI), was performed according to clinical indication and evolving local protocols during the study period. Throughout the study period, IVT was administered as intravenous alteplase at a dose of 0.9 mg/kg (maximum 90 mg) within 4.5 h of symptom onset or of the time the patient was last known to be well; alteplase was the only thrombolytic agent used, and no patient in this cohort received tenecteplase. Patients with an unknown time of onset, including those with wake-up stroke, were treated on the basis of advanced imaging in accordance with the protocols in force at the time. Baseline NIHSS scores were obtained in the emergency department by neurology residents of varying clinical experience as part of the acute stroke pathway; but everyone had completed formal NIHSS certification and participated in the centre’s weekly in situ simulation-based team training. The influence of clinical experience on performance within this pathway at our centre has been reported separately [15].

### Data collection and definitions

Data were obtained from a prospectively maintained local IVT registry including all patients treated with IVT for suspected ischaemic stroke. The registry captures clinical data at admission, during hospitalisation, and at 90-day follow-up. Recorded variables include age, sex, pre-stroke functional status assessed by the modified Rankin Scale (mRS), vascular risk factors, NIHSS scores at admission, 24 h, and discharge, onset-to-needle and door-to-needle times, and 90-day functional outcomes assessed by mRS. sICH was defined according to ECASS II criteria as neurological deterioration of ≥ 4 points on the NIHSS in combination with intracranial haemorrhage on follow-up imaging [16].

Final diagnoses were based on the discharge diagnoses following in-hospital diagnostic evaluation. All patients in whom acute cerebral infarction was suspected underwent MRI for diagnostic confirmation, and every case was reviewed by a consultant stroke neurologist the day after admission. The final diagnosis was assigned by that consultant based on the imaging findings, the clinical history and the complete in-hospital diagnostic work-up. Diagnoses were not adjudicated independently by a second reviewer or by a blinded panel, and stroke mimic status is reported as a descriptive characteristic of the treated population rather than as a treatment outcome. Acute ischaemic stroke was defined as the final clinical diagnosis of stroke, including patients without imaging-confirmed infarction when stroke was considered the most likely diagnosis by the treating team. Stroke mimics were defined as patients treated for suspected stroke but discharged with a non-stroke diagnosis.

Favourable early neurological status was defined as an NIHSS score of 0–1 at 24 h. Functional independence was defined as an mRS score of 0–2 at 90 days, and poor functional outcome as an mRS 3–6 at 90 days.

### Study outcomes

The primary outcomes were favourable early neurological status, defined as NIHSS 0–1 at 24 h, and functional independence, defined as mRS 0–2 at 90 days. Secondary outcomes were poor functional outcome, defined as mRS 3–6 at 90 days, sICH, final diagnosis of stroke mimic, and subgroup comparison between patients with NIHSS 0–2 and NIHSS 3–5 at admission.

### Statistical analyses

Continuous variables were presented as mean with standard deviation or median with interquartile range, as appropriate. Categorical variables were presented as counts and percentages.

Group comparisons between patients with NIHSS 0–2 and NIHSS 3–5 were performed using Student’s t-test or the Mann–Whitney U test for continuous variables, and the Chi-square test for categorical variables.

In patients with confirmed stroke, multivariable binary logistic regression analyses were performed to assess associations between NIHSS subgroup and outcomes, including favourable early neurological status, functional independence, and poor functional outcome. Covariates were selected a priori and included age, sex, hypertension, diabetes, atrial fibrillation, previous stroke or transient ischaemic attack (TIA), hypercholesterolaemia, smoking status, prior antithrombotic use, baseline mRS and onset-to-needle time. Results were reported as odds ratios (OR) with 95% confidence intervals (CI). A two-sided p-value < 0.05 was considered statistically significant. Analyses were performed using SPSS version 26 (IBM Corp., Armonk, NY, USA).

To address temporal changes in imaging, patient selection and acute stroke care over the study period, the cohort was divided into three treatment eras (2008–2014, 2015–2019 and 2020–2023). Baseline characteristics, process metrics and outcomes were compared across eras and tested for linear trend using the Cochran–Armitage test for proportions and the Jonckheere–Terpstra test for continuous variables. Calendar year was subsequently entered as a continuous covariate in the multivariable models as a sensitivity analysis. Patients with and without a recorded 90-day mRS were compared with respect to age, admission NIHSS, sex, and year of treatment in order to assess the potential for attrition bias.

## Results

### Study population

**Fig. 1:**
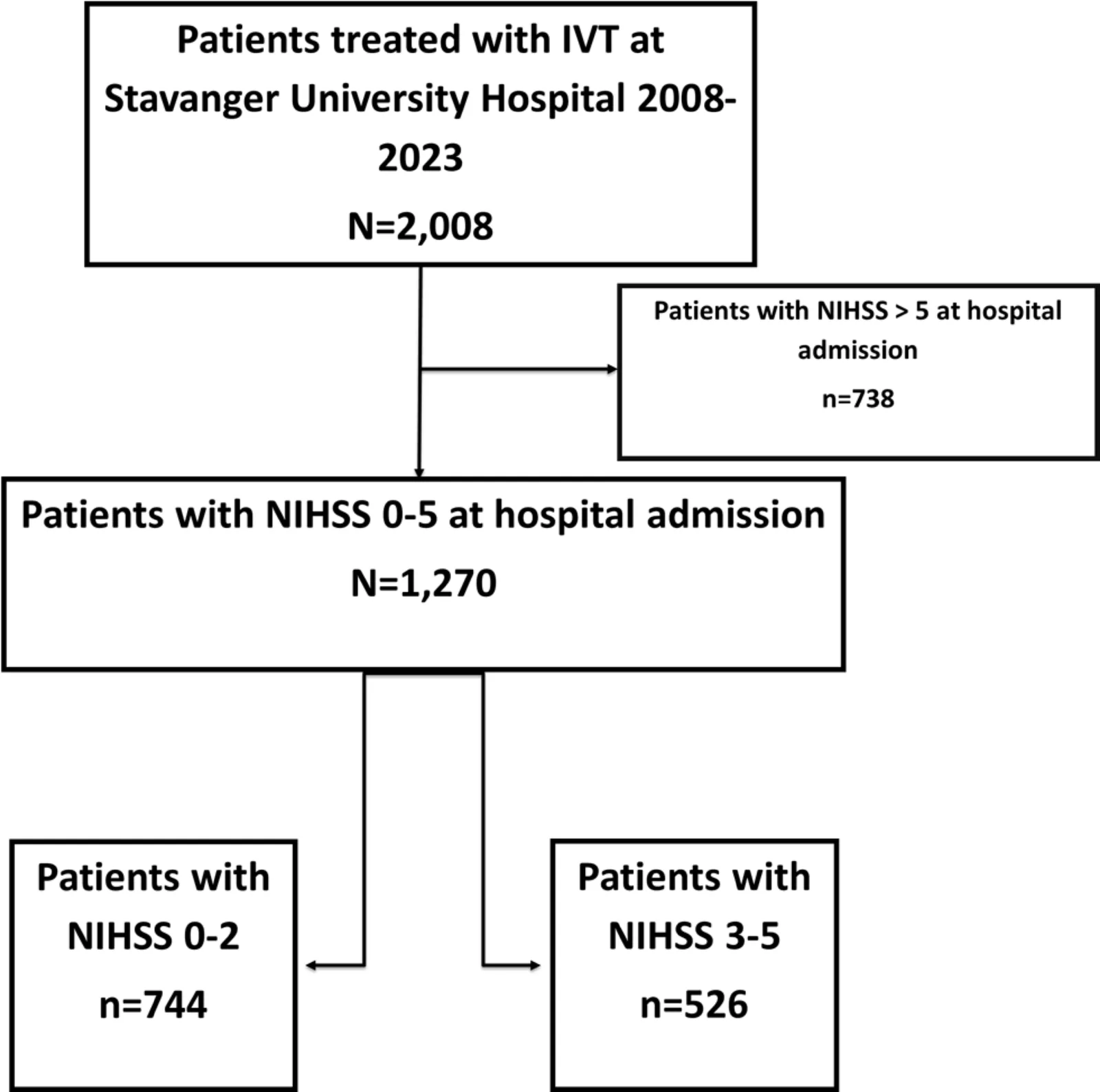
Flowchart of study population. Flowchart depicting patients treated with intravenous thrombolysis (IVT) at Stavanger University Hospital between 2008 and 2023. Of 2,008 IVT-treated patients, 1270 presented with minor neurological deficits (NIHSS 0–5) and were included in the study. Patients were stratified into NIHSS 0–2 and NIHSS 3–5 subgroups based on admission NIHSS score

During the study period, a total of 2,008 patients were treated with IVT at SUH. Of these, 1270 patients presented with minor neurological deficits, defined as admission NIHSS 0–5, and were included in the study. The remaining 738 patients had admission NIHSS > 5 and were excluded. Among included patients, 744 (59%) had NIHSS 0–2 and 526 (41%) had NIHSS 3–5 at hospital admission (Fig. 1). An admission NIHSS of 0 was recorded in 206 patients (16%).

### Baseline characteristics

Baseline characteristics, vascular risk factors, process metrics and outcomes for the total cohort and the two NIHSS subgroups are presented in Table 1. The mean age in the total cohort was 66 years, and patients with NIHSS 0–2 were younger than those with NIHSS 3–5 (64 vs. 69 years, *p* < 0.001). Pre-stroke functional status was comparable between the subgroups. Patients with NIHSS 0–2 had a lower burden of vascular comorbidity, with lower rates of atrial fibrillation, hypertension and prior antithrombotic use and a higher mean eGFR, whereas diabetes, smoking status and hypercholesterolaemia did not differ significantly between the groups (Table 1). Median door-to-needle time was 26 min in the total cohort, and neither door-to-needle nor onset-to-needle time differed significantly between the subgroups. Endovascular treatment was performed in 26 patients (2%) and was more frequent in the NIHSS 3–5 group (Table 1).

**Table 1:** Baseline characteristics and clinical outcomes of patients with minor ischaemic stroke (NIHSS 0–5)

Characteristics	Total study population NIHSS 0–5 *N* = 1270	NIHSS 0–2 *N* = 744 (59%)	NIHSS 3–5 *N* = 526 (41%)	*p*-value
Mean age, years	66 (65–67)	64 (63–66)	69 (67–70)	< 0.001
Male sex, %	55	57	52	0.22
Median baseline mRS score	0 (0–0)	0 (0–0)	0 (0–0)	–
Median NIHSS score on admission	2 (1–4)	1 (0–2)	4 (3–4)	< 0.001
**Cardiovascular disease**				
Atrial fibrillation, % (n)	8 (106)	7 (52)	10 (54)	0.038
Hypertension, % (n)	44 (561)	40 (300)	50 (261)	0.0040
Diabetes, % (n)	13 (168)	12 (87)	15 (81)	0.061
Active smoker, n (%)	19 (238)	19 (140)	20 (98)	0.70
Former smoker, n (%)	20 (250)	21 (155)	19 (95)	0.70
**Former stroke or TIA**				
Former stroke or TIA, % (n)	19 (244)	17 (128)	22 (116)	0.036
Use of anticoagulation or antiplatelets prior to admission, % (n)	34 (426)	31 (230)	37 (196)	0.022
Hypercholesterolaemia, % (n)	37 (467)	35 (260)	39 (207)	0.30
**Biomarkers**				
Glucose, mmol/L	7 (7–7)	7 (7–7)	7 (7–7)	0.26
Mean urea, mmol/L	7 (7–7)	7 (6–7)	7 (7–7)	0.016
Mean creatinine, µmol/L	85 (83–86)	81 (80–84)	88 (85–92)	0.001
Mean eGFR, mL/min/1.73 m²	78 (77–80)	81 (80–83)	74 (72–76)	< 0.001
Median baseline troponin, ng/L	4 (4–6)	4 (4–5)	4 (4–5)	< 0.001
**Metrics and treatments**				
Mean onset-to-needle time, minutes	120 (80–176)	140 (131–150)	130 (121–138)	0.102
Thrombectomy, % (n)	2 (26)	1 (8)	4 (18)	0.003
**Outcomes**				
Functional independence (mRS 0–2) at 90 days post-index stroke, % (n)	86 (602/697)	93 (383/410)	76 (219/287)	< 0.001
sICH, n (%)	4 (< 1)	3 (< 1)	1 (< 1)	0.364
Stroke mimics, % (n)	26 (335)	29 (212)	23 (123)	0.041

Baseline characteristics and clinical outcomes in patients with minor ischaemic stroke (NIHSS 0–5) treated with intravenous thrombolysis and stratified by baseline NIHSS score. Data are presented as mean (95% confidence interval), median (interquartile range), or number (percentage), as appropriate. P-values represent comparisons between patients with NIHSS 0–2 and NIHSS 3–5 at admission. Functional outcomes are reported among patients with available 90-day follow-up. Abbreviations: NIHSS, National Institutes of Health Stroke Scale; mRS, modified Rankin Scale; TIA, transient ischaemic attack; eGFR, estimated glomerular filtration rate; sICH, symptomatic intracranial haemorrhage

Among the 26 patients treated with endovascular thrombectomy, the median age was 73 years (IQR 59–78) and the median admission NIHSS was 3 (IQR 2–4, range 0–5); four patients (15%) had an admission NIHSS of 0. The site of occlusion was documented in the registry for four patients (right M1, left M1, right M1 and left P1) and was not captured as a structured variable for the remainder. The indication for thrombectomy was likewise not recorded in a structured format; according to the documentation of the treating clinicians, the predominant indication was neurological deterioration after admission rather than primary thrombectomy for a mild deficit with a known occlusion. No patient in this subgroup sustained sICH. A 90-day mRS was available for 22 of the 26 patients, of whom 15 (68%) achieved mRS 0–2 and 7 (32%) mRS 3–6, including two deaths.

### Final diagnosis and stroke mimics

A final diagnosis of stroke mimic was recorded in 335 patients, corresponding to 26% of the total cohort. Stroke mimics were more frequent among patients with NIHSS 0–2 than among those with NIHSS 3–5 (29% vs. 23%, *p* = 0.041).

### Functional outcomes

**Fig. 2:**
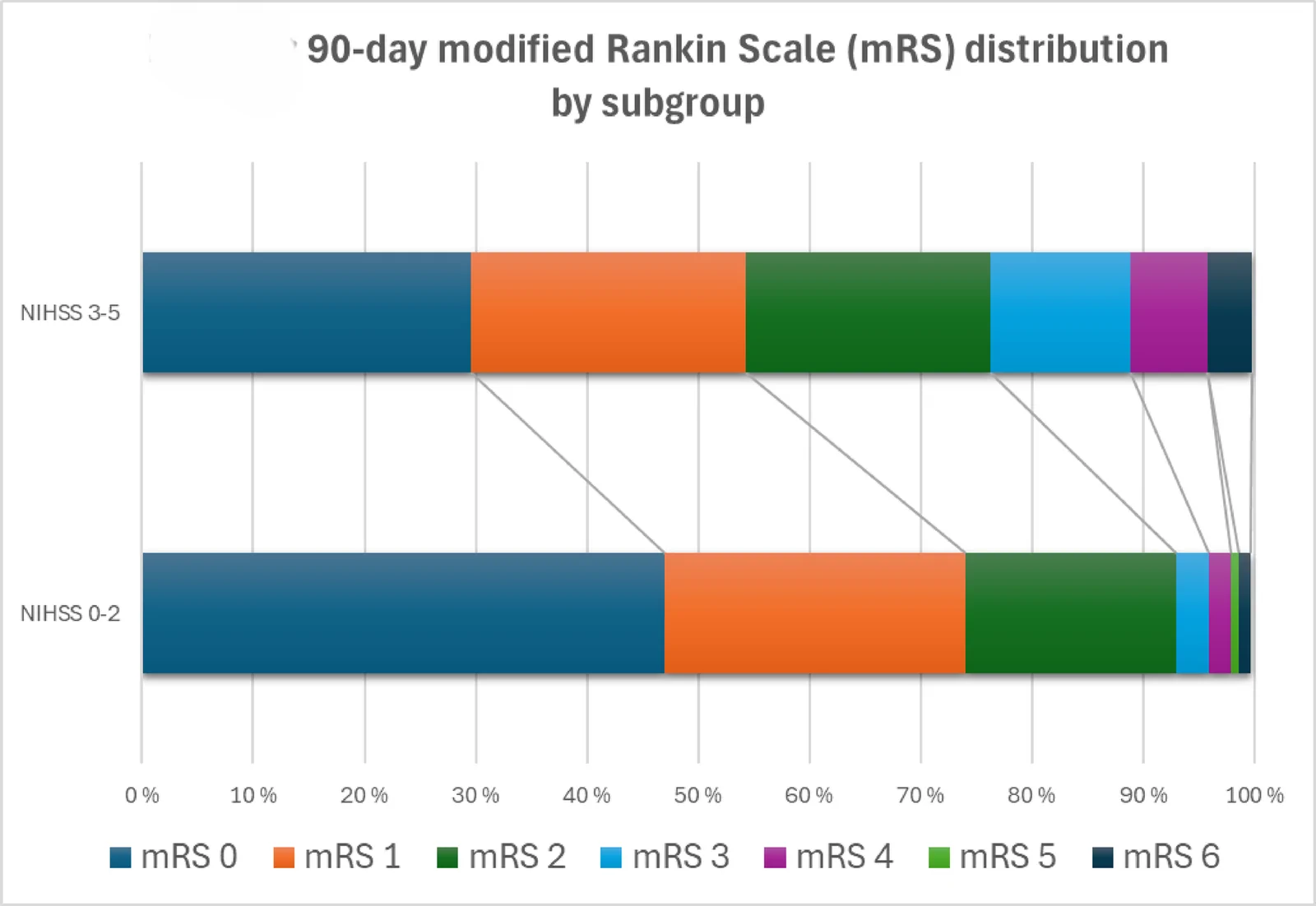
90-day modified Rankin Scale (mRS) distribution by subgroup. Stacked bar chart presenting the distribution of 90-day mRS scores (0–6) among patients with confirmed ischaemic stroke treated with intravenous thrombolysis, stratified by baseline NIHSS (0–2 vs. 3–5). A higher proportion of patients with NIHSS 0–2 achieved functional independence (mRS 0–2) compared with those with NIHSS 3–5

A 90-day mRS was not collected in patients with a final diagnosis of stroke mimic, and the analysis of functional outcome was therefore restricted to patients with a final diagnosis of stroke. A 90-day mRS was available in 697/935 confirmed stroke patients, corresponding to a 75% coverage rate. Patients with an available 90-day mRS were slightly older than those without (median 72 versus 69 years, *p* = 0.042) and had a marginally lower admission NIHSS (median 2, IQR 1–3, versus median 2, IQR 1–4; *p* = 0.021), and they were treated later in the study period (median year of treatment 2018 versus 2013, *p* < 0.001); the proportion of women was similar in the two groups (41% versus 42%). Among confirmed stroke patients with available follow-up, 602 achieved functional independence at 90 days, corresponding to 86%. Functional independence was more frequent in the NIHSS 0–2 group than in the NIHSS 3–5 group (383/410, 93% vs. 219/287, 76%; *p* < 0.001). The distribution of 90-day mRS by NIHSS subgroup is presented in Fig. 2.

### Safety outcomes

sICH occurred in four patients overall, corresponding to < 1% of the total cohort. Three events occurred in the NIHSS 0–2 group and one event occurred in the NIHSS 3–5 group, with no significant difference between groups. No cases of sICH occurred among patients with a final diagnosis of stroke mimic.

### Adjusted subgroup analyses

Multivariable binary logistic regression analyses were performed in patients with confirmed stroke, excluding stroke mimics. After adjustment for prespecified covariates, patients with admission NIHSS 0–2 had higher odds of favourable early neurological status compared with patients with NIHSS 3–5 (adjusted OR 3.76, 95% CI 2.27–6.23; Table 2a).

**Table 2:** a Multivariable logistic regression analysis of favourable early neurological status (NIHSS 0–1 at 24 h), NIHSS 0–2 vs. NIHSS 3–5

Variable	Adjusted OR	95% CI
**Early neurological improvement (main exposure: NIHSS 0–2 vs. 3–5)**	**3.76**	**2.27–6.23**
Age	0.99	0.97–1.01
Female sex	1.15	0.70–1.90
Atrial fibrillation	0.68	0.33–1.43
Hypertension	0.90	0.54–1.51
Diabetes	0.61	0.32–1.19
Active smoker	0.94	0.50–1.76
Former stroke or TIA	0.89	0.47–1.72
Use of anticoagulation or antiplatelets prior to admission	1.35	0.69–2.63
Hypercholesterolaemia	1.46	0.75–2.82
Baseline mRS	0.69	0.53–0.91
Onset-to-needle time, minutes	0.998	0.996–1.000

Multivariable logistic regression analysis of factors associated with favourable early neurological status (NIHSS 0–1 at 24 h) in patients with confirmed ischaemic stroke. Odds ratios (ORs) with 95% confidence intervals (CIs) are presented. The main exposure is baseline NIHSS 0–2 compared with NIHSS 3–5. Analyses were adjusted for prespecified covariates including age, sex, vascular risk factors, prior antithrombotic use, baseline mRS and onset-to-needle time, and were restricted to patients with confirmed stroke, excluding stroke mimics (*n* = 637). Abbreviations: OR, odds ratio; CI, confidence interval; NIHSS, National Institutes of Health Stroke Scale; mRS, modified Rankin Scale; TIA, transient ischaemic attack

**Table 3:** b Multivariable logistic regression analysis of functional independence (mRS 0–2 at 90 days), NIHSS 0–2 vs. NIHSS 3–5

Variable	Adjusted OR	95% CI
**Functional independence (main exposure: NIHSS 0–2 vs. 3–5)**	**3.57**	**1.85–6.89**
Age	0.96	0.94–0.99
Female sex	1.11	0.58–2.14
Atrial fibrillation	0.52	0.22–1.24
Hypertension	1.11	0.57–2.16
Diabetes	0.30	0.13–0.66
Active smoker	0.99	0.41–2.40
Former stroke or TIA	1.24	0.55–2.83
Use of anticoagulation or antiplatelets prior to admission	0.66	0.29–1.50
Hypercholesterolaemia	1.71	0.75–3.91
Baseline mRS	0.24	0.16–0.35
Onset-to-needle time, minutes	1.001	0.997–1.005

Multivariable logistic regression analysis of factors associated with functional independence (mRS 0–2 at 90 days) in patients with confirmed ischaemic stroke. Odds ratios (ORs) with 95% confidence intervals (CIs) are presented. The main exposure is baseline NIHSS 0–2 compared with NIHSS 3–5. Analyses were adjusted for prespecified covariates including age, sex, vascular risk factors, prior antithrombotic use, baseline mRS and onset-to-needle time, and were restricted to patients with confirmed stroke and available 90-day follow-up (*n* = 625). Because mRS 0–2 and mRS 3–6 are complementary outcomes in the same patients, every estimate in Table 4c is the reciprocal of the corresponding estimate in Table 3b. Abbreviations: OR, odds ratio; CI, confidence interval; NIHSS, National Institutes of Health Stroke Scale; mRS, modified Rankin Scale; TIA, transient ischaemic attack

Similarly, NIHSS 0–2 was associated with higher odds of functional independence at 90 days compared with NIHSS 3–5 (adjusted OR 3.57, 95% CI 1.85–6.89; Table 3b). Poor functional outcome, defined as mRS 3–6 at 90 days, was recorded in 27/410 patients (7%) in the NIHSS 0–2 group and in 68/287 patients (24%) in the NIHSS 3–5 group. Poor functional outcome was correspondingly less frequent in the NIHSS 0–2 group (adjusted OR 0.28, 95% CI 0.15–0.54; Table 4c).

Patient characteristics and process metrics changed over the study period, whereas outcomes did not (Supplementary Table S1). Median admission NIHSS fell from 3 to 2 and the proportion of stroke mimics rose from 7% to 38% (both p for trend < 0.001), consistent with a progressively lower treatment threshold. The proportion of women rose from 38% to 52% (p for trend < 0.001). Median door-to-needle time fell from 40 min in 2008–2014 to 20 min in 2015–2019 but rose to 23 min in 2020–2023 (p for trend < 0.001). Functional outcomes appeared stable, with 88%, 88% and 85% of patients achieving mRS 0–2 (p for trend = 0.26) and no trend in mortality (p for trend = 0.89). No 90-day mRS was recorded before 2014 and coverage fell from 84% in 2014 to 45% in 2023. The four sICH events occurred in 2010, 2012 and 2021 and showed no temporal pattern.

**Table 4:** c Multivariable logistic regression analysis of poor functional outcome (mRS 3–6 at 90 days), NIHSS 0–2 vs NIHSS 3–5

Variable	Adjusted OR	95% CI
**Poor functional outcome, mRS 3–6 (main exposure: NIHSS 0–2 vs 3–5)**	**0.28**	**0.15–0.54**
Age	1.04	1.01–1.07
Female sex	0.90	0.47–1.72
Atrial fibrillation	1.91	0.80–4.52
Hypertension	0.90	0.46–1.75
Diabetes	3.36	1.52–7.46
Active smoker	1.01	0.42–2.43
Former stroke or TIA	0.81	0.35–1.83
Use of anticoagulation or antiplatelets prior to admission	1.51	0.67–3.40
Hypercholesterolaemia	0.58	0.26–1.33
Baseline mRS	4.17	2.82–6.16
Onset-to-needle time, minutes	0.999	0.995–1.003

Multivariable logistic regression analysis of factors associated with poor functional outcome (mRS 3–6 at 90 days) in patients with confirmed ischaemic stroke. Odds ratios (ORs) with 95% confidence intervals (CIs) are presented. The main exposure is baseline NIHSS 0–2 compared with NIHSS 3–5. Analyses were adjusted for prespecified covariates including age, sex, vascular risk factors, prior antithrombotic use, baseline mRS and onset-to-needle time, and were restricted to patients with confirmed stroke and available 90-day follow-up (n=625). Because mRS 0–2 and mRS 3–6 are complementary outcomes in the same patients, every estimate in Table 4c is the reciprocal of the corresponding estimate in Table 3b. Abbreviations: OR, odds ratio; CI, confidence interval; NIHSS, National Institutes of Health Stroke Scale; mRS, modified Rankin Scale; TIA, transient ischaemic attack

## Discussion

In this large real-world cohort of IVT-treated patients presenting with minor ischaemic stroke symptoms, we found very low rates of symptomatic intracranial haemorrhage, with no apparent safety signal between patients with NIHSS 0–2 vs. 3–5. Functional outcomes were favourable in both subgroups, although patients with very mild deficits had higher rates of early neurological recovery and functional independence at 90 days. This difference is not unexpected, as baseline NIHSS is a well-established predictor of post-stroke outcome [17, 18]. Stroke mimics were more frequent among patients with NIHSS 0–2, reflecting the diagnostic uncertainty associated with mild acute neurological symptoms [19]. sICH remained rare overall, and no events occurred among patients with a final diagnosis of stroke mimic. These findings must, however, be read as a description of a treated cohort rather than as evidence of a treatment effect [20]. Because every patient in this cohort received IVT, and because the decision to treat reflected clinical judgement at the bedside, the design can address the safety of the strategy but cannot establish its efficacy.

Our findings are consistent with observational studies suggesting that IVT in minor ischaemic stroke is safe and may be associated with beneficial functional outcomes. A Danish nationwide cohort reported low rates of sICH (1%), favourable outcomes in IVT-treated mild strokes, and lower 1-year mortality rates both in mild and very mild strokes [21]. Similarly, a large meta-analysis including over 90,000 patients reported no increase in sICH rates, higher rates of early neurological improvement, and excellent functional outcome (mRS 0–1) [13]. These findings support that IVT is associated with low complication risks and that IVT should continue to be evaluated in carefully selected patients with mild deficits.

Randomised trials have yielded different results, but they enrolled populations that differ materially from ours and from one another. A direct comparison with the present cohort is therefore not appropriate. PRISMS compared alteplase with aspirin within three hours of onset in patients with non-disabling deficits [7]; ARAMIS compared alteplase with DAPT [10]; and TEMPO-2 randomised patients with a proven intracranial occlusion or a favourable perfusion profile in a 4.5–12-hour window [11]. Because the three trials differ from one another and from our cohort in comparator, time window and eligibility, we do not set our outcome rates against theirs.

In the randomised trials, rates of excellent functional outcome (mRS 0–1) were high. This may have limited the ability to detect any meaningful clinical difference in outcomes using the mRS. The scale has been criticised for its low sensitivity in identifying post-stroke disability [6, 22]. Disabilities such as cognitive dysfunction, fatigue, speech disturbances, impairments in executive function and social participation may persist despite an mRS score of 0–2. In response to this, new post-stroke clinical assessment and patient-related outcome measurement tools are in development, such as the Canadian Outcome Scale for Minor Stroke (COSMOS) [23]. These limitations apply directly to our own outcome measures. In patients whose admission NIHSS is already 0–2, the scope for measurable improvement to an NIHSS of 0–1 is minimal, and the comparison of NIHSS 0–2 with NIHSS 3–5 for this endpoint is influenced by proximity to the outcome threshold at baseline as much as by any treatment effect. We nonetheless report this endpoint, because it is a standardised and widely used measure, and because an NIHSS of 0–1 at 24 h documents the absence of early neurological deterioration, which is itself clinically relevant in a population in which an occlusion may persist. The same reasoning applies to the mRS: with 86% of our patients reaching mRS 0–2, the scale has little room in which to discriminate, and a high rate of functional independence in a minor stroke cohort should not be read as evidence for IVT benefit. Any discrepancy between the findings of observational cohorts and those of randomised trials is likely multifactorial. It may reflect factors such as broader inclusion criteria in observational studies, challenges in defining disabling symptoms, variation in real-world treatment implementation and time-to-treatment metrics, and the risk of clinical deterioration despite initial mild symptom presentation [24].

A central unresolved issue remains the distinction between disabling and non-disabling symptoms [12]. The newest guidelines on acute ischaemic stroke management by the American Heart Association emphasise this by recommending treatment with IVT in minor disabling stroke [25]. Yet, determining whether symptoms are truly disabling in the acute setting remains a challenge. Currently, there are no standardised definitions of disabling symptoms. An individualised assessment in each case, based on patient characteristics, baseline function and occupational status, is therefore still needed.

Another consideration is the time-sensitive nature of the comparator treatment. The efficacy of DAPT appears greatest when treatment is initiated very early after symptom onset, usually under protocol-driven trial conditions [26]. In routine clinical practice, diagnostic uncertainty, imaging workflows, and organisational factors may delay treatment initiation. Such delays may attenuate the effectiveness of DAPT and limit the generalisability of trial-based non-inferiority findings to everyday stroke care.

The proportion of stroke mimics treated in this cohort also carries economic implications. In a decision-analytic model, thrombolysis of suspected stroke within three hours remained cost-effective at a willingness-to-pay threshold of US$100 000 per quality-adjusted life-year up to a stroke mimic proportion of approximately 30%, above which it was no longer cost-effective [27]. Our overall mimic proportion of 26% lies just below that threshold, but it rose from 7% in the earliest era to 38% in the most recent one, and reached 46% among patients with an admission NIHSS of 0. Since the present data cannot demonstrate a benefit of IVT over no treatment, we are not in a position to argue that the associated drug, monitoring and complication costs are justified relative to DAPT, and a rising mimic proportion is a legitimate reason to keep the economic consequences of a liberal policy under review. We have previously reported that faster treatment at this centre was accompanied by an increase in the proportion of treated patients subsequently diagnosed with peripheral vertigo, with no sICH in that group [28]; the safety argument and the economic argument therefore point in different directions and should be weighed separately.

Our cohort adds real-world safety data from a large consecutive population treated in a centre with a historically liberal thrombolysis policy. While these findings should not be interpreted as evidence of treatment efficacy, they underscore the need for improved patient phenotyping and more sensitive outcome measures in future minor stroke studies [29]. Hence, the question of whether to offer thrombolysis to patients with mild or non-disabling stroke symptoms continues to exist, and the evidence required to resolve it will have to come from randomised comparisons. For the subgroup with an intracranial occlusion and mild deficits, that evidence is awaited from the ongoing ENDOLOW (NCT04167527) and MOSTE (NCT03796468) trials.

This study has several important limitations. First, the retrospective observational design introduces a risk of residual confounding and limits causal inference. Second, the inclusion of data spanning a 16-year period may have introduced bias due to temporal changes in clinical practice, diagnostic criteria and acute stroke management; we have explored this by stratifying the cohort into treatment eras and by adjusting for calendar year, but unmeasured secular change cannot be excluded. Third, the absence of an untreated control group precludes direct comparison between IVT and dual antiplatelet therapy or no treatment, and the allocation of patients to thrombolysis reflected clinical judgement, which is in itself a source of selection bias. Our findings should therefore be interpreted as descriptive of outcomes and safety rather than efficacy. Fourth, the early neurological improvement outcome measure was subject to ceiling effects, particularly in the NIHSS 0–2 group, which may have overestimated early neurological improvement in this group. Fifth, 90-day mRS was available for only 75% of confirmed stroke patients, and those lost to follow-up differed with respect to age, admission NIHSS and year of treatment, so that attrition bias cannot be excluded. Sixth, the distinction between disabling and non-disabling deficits was not recorded systematically and cannot be reconstructed from the total NIHSS score alone. This is a substantial limitation, since it is precisely this distinction on which current guideline recommendations turn. Seventh, although CT angiography was performed as part of routine care, the presence and site of intracranial occlusion were not entered as structured registry variables, and we are therefore unable to describe outcomes according to occlusion status or to characterise the indication for thrombectomy beyond the documentation of the treating clinicians. Eighth, recurrent stroke and transient ischaemic attack within 90 days were not captured in the registry and could not be reported. Ninth, only four sICH events occurred; this is reassuring descriptively, but the number of events is too small to support any firm conclusion regarding the safety of a liberal thrombolysis strategy, and the confidence interval around the estimate is correspondingly wide. Finally, assessment of symptom severity and disability relied on routine clinical documentation and may be subject to misclassification.

## Conclusions

In an unselected real-world cohort, IVT-treated patients with NIHSS 0–5 achieved favourable functional outcomes and very low complication rates. These findings support continued evaluation of IVT in carefully selected patients with mild deficits. The discrepancies between real-world outcomes and randomised trials highlight the limitations of current patient selection strategies and outcome measures. These findings provide clinically relevant safety data but should be interpreted descriptively in the absence of an untreated comparator group. Future research should focus on refining definitions of disabling symptoms, accounting for timing of DAPT, improving risk stratification for early neurological deterioration, and incorporating more sensitive functional and patient-reported outcome measures.

## Supplementary Information

Supplementary Material 1.

## Data Availability

The datasets analysed during the current study are not publicly available due to national data protection regulations and the terms of the ethics approval, which restrict sharing of individual-level patient data. De-identified data may be made available from the corresponding author upon reasonable request, subject to approval from the Western Norway Regional Ethics Committee and completion of a data processing agreement.
