# Supplementary material for: Outcomes and safety of intravenous thrombolysis in minor ischaemic stroke: a real-world retrospective cohort study
Source: Neurol Res Pract. 2026 Nov 10;8(1):95. doi: 10.1186/s42466-026-00552-x (PMC13753290; doi:10.1186/s42466-026-00552-x)
Supplement: Supplementary file 1 [file 42466_2026_552_MOESM1_ESM.docx]

**Supplementary Material**

**Supplementary Table S1.** Patient characteristics, process metrics and outcomes by treatment era.

|  | **2008–2014**  **(n=207)** | **2015–2019**  **(n=532)** | **2020–2023**  **(n=531)** | **p for trend** |
| --- | --- | --- | --- | --- |
| **Patient characteristics** |  |  |  |  |
| Age, years, median (IQR) | 69 (58–80) | 67 (54–78) | 70 (56–78) | 0.76 |
| Female, n (%) | 78/207 (37.7) | 221/532 (41.5) | 270/524 (51.5) | <0.001 |
| NIHSS on admission, median (IQR) | 3 (2–4) | 2 (1–3) | 2 (1–3) | <0.001 |
| Wake-up stroke, n (%) | 8/71 (11.3) | 44/532 (8.3) | 32/488 (6.6) | 0.49 |
| Stroke mimic, n (%) | 14/207 (6.8) | 129/532 (24.2) | 192/502 (38.2) | <0.001 |
| **Process metrics and treatment** |  |  |  |  |
| Door-to-needle time, min, median (IQR) | 40 (29–61) | 20 (13–34) | 23 (15–40) | <0.001^a^ |
| Onset-to-needle time, min, median (IQR) | 134 (94–184) | 117 (78–170) | 126 (89–195) | 0.19^a^ |
| Endovascular thrombectomy, n (%) | 4/192 (2.1) | 9/530 (1.7) | 13/530 (2.5) | 0.97 |
| **Outcomes** |  |  |  |  |
| mRS 0–2 at 90 days, n (%) | 50/57 (87.7) | 312/356 (87.6) | 240/284 (84.5) | 0.26^b^ |
| Death at 90 days (mRS 6), n (%) | 1/57 (1.8) | 8/356 (2.2) | 7/284 (2.5) | 0.89^b^ |
| Symptomatic intracranial haemorrhage, n (%) | 2/207 (1.0) | 0/532 (0) | 2/531 (0.4) | NA^c^ |

Patients with minor ischaemic stroke (NIHSS 0–5) treated with intravenous thrombolysis, grouped into three treatment eras to describe temporal changes in patient selection, treatment workflow, safety and outcomes. Denominators vary because of missing data. Trend across eras was tested with the Cochran–Armitage test for proportions and the Jonckheere–Terpstra test for continuous variables.

^a^ Door-to-needle and onset-to-needle times were derived from the recorded arrival and bolus times (stroke chart, admission note, emergency record or electronic record, in that order of priority). This derivation reproduces the times already held in the registry for 2008–2019 in 96% (door) and 99.6% (needle) of cases. Door-to-needle time fell to a nadir of 15–16 minutes in 2017–2019 and rose again thereafter, so the trend test understates a non-monotone pattern (2015–2019 versus 2020–2023, p=0.001). Onset-to-needle time showed no trend across the study period (2008–2014 versus 2020–2023, p=0.81).

^b^ No 90-day mRS was recorded before 2014, and coverage fell from 84% in 2014 to 45% in 2023 (697 of 1,270 patients overall). The outcome comparisons therefore cover 2014–2023 and rest on a shrinking and increasingly selected denominator.

^c^ No trend test is reported for symptomatic intracranial haemorrhage because only four events occurred.

*Abbreviations:* IQR, interquartile range; NIHSS, National Institutes of Health Stroke Scale; mRS, modified Rankin Scale; NA, not applicable.
